# Some Optimal Conditions for the ASCLT

**DOI:** 10.1007/s10959-023-01245-w

**Published:** 2023-05-06

**Authors:** István Berkes, Siegfried Hörmann

**Affiliations:** 1 A. Rényi Institute of Mathematics, Reáltanoda u. 13-15, Budapest, 1053 Hungary; 2 Institute of Statistics, Graz University of Technology, Kopernikusgasse 24/III, 8010 Graz, Austria

**Keywords:** Sums of independent random variables, Almost sure central limit theorem, Weighted averages, 60F05, 60F15, 60G50

## Abstract

Let $X_{1},X_{2},\ldots$ be independent random variables with ${E}X_{k}=0$ and $\sigma _{k}^{\,2}:={E}X_{k}^2<\infty$ $(k\ge 1)$. Set $S_k=X_1+\cdots +X_k$ and assume that $s_{k}^{\,2}:={E}S_k^2\rightarrow \infty$. We prove that under the Kolmogorov condition

1 $$\begin{aligned} |X_n|\le L_n, \quad L_n =o(s_n/(\log \log s_n)^{1/2}) \end{aligned}$$

we have

2 $$\begin{aligned} \frac{1}{\log s_{n}^{\,2}}\sum _{k=1}^{n}\frac{\sigma _{k+1}^{\,2}}{s_{k}^{\,2}}f\left( \frac{S_{k}}{s_{k}}\right) \rightarrow \frac{1}{\sqrt{2\pi }}\int _{\mathbb {R}}f(x)e^{-x^2/2}\,\textrm{d}x\quad \mathrm {a.s.}\end{aligned}$$

for any almost everywhere continuous function $f: {\mathbb R} \rightarrow {\mathbb R}$ satisfying $|f(x)|\le e^{\gamma x^2}$, $\gamma <1/2$. We also show that replacing the *o* in (1) by *O*, relation (2) becomes generally false. Finally, in the case when (1) is not assumed, we give an optimal condition for (2) in terms of the remainder term in the Wiener approximation of the partial sum process $\{S_n, \, n\ge 1\}$ by a Wiener process.

## Introduction

Let $X_1,X_2,\ldots$ be i.i.d. random variables with ${E}X_1=0$, ${E}X_1^2=1$ and put $S_n=X_1+\cdots +X_n$. In its simplest form, the almost sure central limit theorem states that for any $x \in \mathbb R$

3 $$\begin{aligned} \lim _{n\rightarrow \infty } \frac{1}{\log n }\sum _{k=1}^n \frac{1}{k} I\left\{ \frac{S_k}{\sqrt{k}}\le x\right\} = \Phi (x) \quad \text {a.s.}, \end{aligned}$$

where *I* denotes indicator function and $\Phi (x)$ is the standard normal distribution function. Relation (3) was proved by Brosamler [7] and Schatte [17] under slightly stronger moment assumptions and by Lacey and Philipp [11] under finite variances.

In past decades, a wide theory of a.s. limit theorems of the type (3) has been developed and several extensions and improvements in (3) were proved. In particular, it turned out that under mild moment assumptions (see (5)), any weak limit theorem

$$\begin{aligned} (S_n-a_n)/b_n \overset{d}{\longrightarrow }\ G \end{aligned}$$

for partial sums $S_n$ of independent random variables $X_1, X_2, \ldots$ has an almost sure version

4 $$\begin{aligned} \lim _{n\rightarrow \infty } \frac{1}{D_n}\sum _{k=1}^n d_k I\left\{ \frac{S_k-a_k}{b_k}\le x\right\} = G(x) \quad \text {a.s.} \end{aligned}$$

where the weights $d_n$ are determined by the norming sequence $(b_n)$ and $D_n=\sum _{k=1}^n d_k$. For $(b_n)$ growing with polynomial speed we have $d_n=1/n$ (see Berkes and Dehling [6]); assuming only $b_n\uparrow \infty$, (4) holds with $d_k= (b_{k+1}-b_k)/b_k$ (Ibragimov and Lifshits [9], Lifshits [12]). The last paper also proves the optimality of the moment assumption

5 $$\begin{aligned} \sup _n {E}\left( \log \log \Big |\frac{S_n-a_n}{b_n} \Big | \right) ^{1+\delta } <\infty \end{aligned}$$

assumed in [6, 12], showing the surprising role of loglog moments in ASCLT theory.

In particular, if $X_1, X_2, \ldots$ are independent random variables with ${E}X_n=0$, $\sigma _n^2={E}X_n^2<\infty$, $s_n^2={E}S_n^2$ and $(X_n)$ satisfies the Lindeberg condition

$$\begin{aligned} \lim _{n\rightarrow \infty } \frac{1}{s_n^2}\sum _{k=1}^n E X_k^2 I_{ \{|X_k|\ge \varepsilon s_k \}}=0 \quad \text {for any} \ \varepsilon >0 \end{aligned}$$

then we have

6 $$\begin{aligned} \lim _{n\rightarrow \infty } \frac{1}{\log s_n^2 }\sum _{k=1}^n \frac{\sigma _{k+1}^2}{s_k^2} I\left\{ \frac{S_k}{s_k}\le x\right\} = \Phi (x) \quad \text {a.s.} \end{aligned}$$

See Atlagh [1], Atlagh and Weber [2] and Major [15]. Formally, the weights in (6) are different from those in (3), but letting $\{S(t), \, t\ge 0\}$ denote the partial sum process defined by

7 $$\begin{aligned} S(t)=S_k \quad \text {for} \quad s_k^2\le t <s_{k+1}^2 \quad (k=0, 1, \ldots ), \end{aligned}$$

relation (6) implies

$$\begin{aligned} \lim _{T\rightarrow \infty } \frac{1}{\log T} \int _1^T \frac{1}{t} I \left\{ \frac{S(t)}{\sqrt{t}} \le x\right\} \, dt =\Phi (x) \quad \text {a.s.} \end{aligned}$$

and thus, we have again an a.s. limit theorem involving logarithmic averages. For critical weight sequences in ASCLT theory, see Hörmann [8]. For an extension for nonlinear limit theorems of the form

$$\begin{aligned} f_n(X_1, X_2, \ldots , X_n)\overset{d}{\longrightarrow }\ G \end{aligned}$$

see Berkes and Csáki [4].

A generalization of (3) in another direction was given by Schatte [18], who proved that if $X_1, X_2, \ldots$ are i.i.d. random variables with ${E}X_1=0$, ${E}X_1^2=1$, ${E}|X_1|^3<\infty$ and $f: {\mathbb R}\rightarrow {\mathbb R}$ is a function almost everywhere continuous with respect to the Lebesgue measure satisfying

8 $$\begin{aligned} |f(x)|\le e^{\gamma x^2}, \quad \gamma <1/4, \end{aligned}$$

then letting $S_n=\sum _{k=1}^n X_k$ we have

9 $$\begin{aligned} \frac{1}{\log n} \sum _{k=1}^{n}\frac{1}{k} f\left( \frac{S_{k}}{\sqrt{k}}\right) \rightarrow \int _{\mathbb {R}}f(x) e^{-x^2/2} dx \quad \text {a.s.} \end{aligned}$$

Berkes, Csáki and Horváth [5] showed that $\gamma <1/4$ in (8) can be replaced by $\gamma <1/2$ and showed also that the assumption ${E}|X_1|^3<\infty$ can be dropped. Ibragimov and Lifshits [10] proved that the finiteness of the integral in (9) plus the assumption that $f(x) e^{-Hx^2}$ is nonincreasing for some $H>0$ is sufficient in (9); on the other hand, the finiteness of the integral itself is not, even if *f* is continuous and $P(X_1=1)=P(X_1=-1)=1/2$. The purpose of the present paper is to study the problem for independent, not necessarily identically distributed random variables with finite variances and to give an optimal condition for the ASCLT. More precisely, we will prove the following theorem.

### Theorem 1

Let $X_{1},X_{2},\ldots$ be independent random variables with ${E}X_{n}=0$ and ${E}X_{n}^2=:\sigma _{n}^{\,2}<\infty$. Set $S_n=X_1+\cdots +X_n$ and $s_{n}^{\,2}:={E}S_n^2$. Assume that $s_{n}^{\,2}\rightarrow \infty$ and

10 $$\begin{aligned} |X_{k}|\le L_{k}, \quad L_{k}=o\left( \frac{s_{k}}{(\log \log s_{k}^{\,2})^{1/2}}\right) . \end{aligned}$$

Then, we have

11 $$\begin{aligned} \frac{1}{\log s_{n}^{\,2}}\sum _{k=1}^{n}\frac{\sigma _{k+1}^{\,2}}{s_{k}^{\,2}}f\left( \frac{S_{k}}{s_{k}}\right) \rightarrow \frac{1}{\sqrt{2\pi }}\int _{\mathbb {R}}f(x)e^{-x^2/2}\,\textrm{d}x\quad \mathrm {a.s.}\quad (n\rightarrow \infty ) \end{aligned}$$

for any almost everywhere continuous function $f:\mathbb {R}\rightarrow \mathbb {R}$ satisfying

12 $$\begin{aligned} |f(x)|\le e^{\gamma x^2}\quad \text {for some }\gamma <1/2. \end{aligned}$$

If (10) only holds with *O* instead of *o*, then (11) is generally false.

In terms of the bounds for the random variables $X_n$, Theorem 1 provides an optimal condition for the ASCLT (11). Note that condition (10) is Kolmogorov’s classical condition for the LIL

13 $$\begin{aligned} \limsup _{n\rightarrow \infty }\, (2s_n^2 \log \log s_n^2)^{-1/2} S_n=1 \quad \text {a.s.} \end{aligned}$$

(see, e.g., [13], p. 272) which, in view of the results of Marcinkiewicz and Zygmund [16] and Weiss [20], is also optimal. This indicates a strong connection between the LIL and ASCLT, a connection which will become very clear by the proof of Theorem 1. As we will see, however, the LIL (13) itself does not imply (11) and neither does the strong approximation

14 $$\begin{aligned} S(t)=W(t) + o(t \log \log t)^{1/2} \quad \text {a.s.} \quad (t\rightarrow \infty ) \end{aligned}$$

where *W* is a Wiener process defined on the same probability space as the sequence $(X_n)$ and $\{S(t), t\ge 0\}$ is the partial sum process defined by (7). The approximation (14) was proved by Strassen [19] in case of i.i.d. sequences $(X_n)$ with mean 0 and variance 1, and he showed that it implies a large class of refinements of the LIL. Major [14] proved that (14) holds also under the Kolmogorov condition (10). As we will see, in the case when (10) does not hold, the validity of (11) is closely connected with the order of Wiener approximability of the partial sums $S_n$. We will namely prove the following results.

### Theorem 2

Let $X_{1},X_{2},\ldots$ be independent, zero mean random variables and define $\sigma _n^2$ and $s_n^2$ as in Theorem 1. Assume that

15 $$\begin{aligned} \sigma _n=o\left( s_n/(\log \log s_n^2)^{1/2}\right) \end{aligned}$$

and there exists a Wiener process *W* such that

16 $$\begin{aligned} S(t)=W(t) + o(t^{1/2}) \quad \text {a.s.} \quad (t\rightarrow \infty ). \end{aligned}$$

Then, for any a.e. continuous function $f: {\mathbb R} \rightarrow {\mathbb R}$ satisfying (12) we have (11). On the other hand, replacing (16) with

17 $$\begin{aligned} S(t)=W(t) + o(t^{1/2}\psi (t)) \quad \text {a.s.} \quad (t\rightarrow \infty ) \end{aligned}$$

with any fixed function $\psi (n)\rightarrow \infty$, (11) becomes generally false.

As the proof will show, the first half of Theorem 2 remains valid if $f(x) = e^{\max \{x^2/2 - cx, 0\}}$, $c > 0$.

### Theorem 3

Under the setup of Theorem 2, assume that (15) holds and there exists a Wiener process *W* such that

18 $$\begin{aligned} S(t)=W(t) + o\left( t/\log \log t\right) ^{1/2} \quad \text {a.s. as} \ t\rightarrow \infty . \end{aligned}$$

Then (11) holds for any measurable function $f:{\mathbb R}\rightarrow {\mathbb R}$ such that the integral on the right side of (11) is finite and $f(x)=e^{x^2/2} g(x)$, where $\log g(x)$ is uniformly continuous on $\mathbb R$.

## Proof of the theorems

### Proof of Theorem 1

By an argument in Schatte [18], it suffices to prove the theorem in the case $f(x)=e^{\gamma x^2}$, $0<\gamma <1/2$. We first note that by the Kolmogorov exponential bounds (see, e.g., Loève [13], p. 266) the assumptions made on the sequence $(X_n)$ in Theorem 1 imply that given any $\epsilon >0$, $K>0$, we have for $n\ge n_0(\epsilon , K)$

19 $$\begin{aligned} P(S_n/s_n \ge x)\le \exp \left( -\frac{x^2}{2}(1-\epsilon )\right) \quad \hbox {for}\quad 0\le x\le K(\log \log s_{n}^{\,2})^{1/2}. \end{aligned}$$

For $1\le k\le l$, we put

$$\begin{aligned} \xi _k=S_k/s_k, \quad \xi _{k,\,l}=(S_{l}-S_{k})/s_{l}. \end{aligned}$$

For a fixed $\delta >0$, we set

20 $$\begin{aligned} M_k=((2+\delta )\log \log s_{k}^{\,2})^{1/2} \end{aligned}$$

and define the truncated variable

21 $$\begin{aligned} \hat{\xi }_{k}= {\left\{ \begin{array}{ll} &{} \xi _k \quad \text {if} \quad |\xi _k| < M_k \\ &{} M_k \quad \ \text {if} \quad \xi _k \ge M_k \\ &{} -M_k \quad \text {if} \quad \xi _k \le -M_k. \end{array}\right. } \end{aligned}$$

### Lemma 1

There exist a constant $\eta >0$ and an integer $k_0$ such that

22 $$\begin{aligned} \textrm{Cov}\, \bigl (f(\hat{\xi }_k), f(\hat{\xi }_l \bigr )) \le 6 (\log s_l)^{2-\eta } (s_k/s_l)^{1/2} \qquad (k_0\le k<l) \end{aligned}$$

and

23 $$\begin{aligned} {E}(f(\hat{\xi }_k)^2) \le (\log s_k^2)^{1 - \eta } \qquad (k\ge k_0). \end{aligned}$$

### Proof

We follow [10]. Define, similar to (21),

24 $$\begin{aligned} \hat{\xi }_{k,\,l}= {\left\{ \begin{array}{ll} &{} \xi _{k, l} \quad \text {if} \quad |\xi _{k, l}| < M_l \\ &{} M_l \quad \ \text {if} \quad \xi _{k, l} \ge M_l \\ &{} -M_l \quad \text {if} \quad \xi _{k, l} \le -M_l \end{array}\right. } \end{aligned}$$

and put

25 $$\begin{aligned} X = f(\hat{\xi }_l) - f(\hat{\xi }_{k,l}). \end{aligned}$$

Clearly $\xi _l - \xi _{k, l} = \frac{s_k}{s_l} \xi _k$ and thus using the mean value theorem and $f'(x) = 2\gamma x f(x)$, we get

$$\begin{aligned} |X| \le |\hat{\xi }_l - \hat{\xi }_{k,l}| 2\gamma \, M_l f(M_l) \le |\xi _l - \xi _{k, l}|2\gamma \, M_l f(M_l)= \frac{s_k}{s_l}|\xi _k| 2\gamma \, M_l f(M_l). \end{aligned}$$

Thus, the integral of |*X*| on the set $\{|\xi _k| \le \left( s_l/s_k\right) ^{1/2}\}$ is at most

$$\begin{aligned} (s_k/s_\ell )^{1/2}2\gamma \, M_l f(M_l). \end{aligned}$$

On the other hand, $|X|\le 2 f(M_l)$ by (25), and thus the integral of |*X*| on the set $\{|\xi _k| > (s_l/s_k)^{1/2}\}$, whose probability is at most $s_k/s_l$ by the Chebysev inequality, is at most $(s_k/s_l) 2f(M_l)$. Therefore,

26 $$\begin{aligned} {E}|X| \le 3 M_l f(M_l) (s_k/s_l)^{1/2}. \end{aligned}$$

On the other hand, the independence of $\hat{\xi }_k$ and $\hat{\xi }_{k,l}$ implies that

$$\begin{aligned}&{E}f(\hat{\xi }_k) f(\hat{\xi }_l) - {E}f(\hat{\xi }_k) \cdot {E}f(\hat{\xi }_l) \\&\qquad = {E}\bigl (f(\hat{\xi }_k)X\bigr ) - {E}f(\hat{\xi }_k) \cdot {E}X \end{aligned}$$

and thus

$$\begin{aligned} \bigl |\textrm{Cov}\bigl (f(\hat{\xi }_k), f(\hat{\xi }_l)\bigr )\bigr | \le 2 f(M_k) {E}|X|. \end{aligned}$$

Now choosing $\delta$ sufficiently small, we have $(2+\delta )\gamma <1$ and thus $M_l f(M_l)^2 \le (\log s_l)^{2-\eta }$ for some constant $\eta >0$ and $l\ge k\ge k_0$. Together with (26), this yields (22).

Now we prove (23). Let $F_k^+$ denote the distribution function of $|\xi _k|$. Integration by parts and using $f(x)=e^{\gamma x^2}$ yields

$$\begin{aligned}&\int _{|\hat{\xi }_k| \le M_k} f^2 (\hat{\xi }_k)dP=\int _0^{M_k}f^2(x)dF_k^+(x)\\&\qquad =F_k^+(M_k)f^2(M_k)-F_k^+(0)f^2(0)-\int _0^{M_k}F_k^+(x)2f(x)f^\prime (x)dx\\&\qquad \le F_k^+(M_k)f^2(M_k)+\int _0^{M_k}(1-F_k^+(x))2f(x)f^\prime (x)dx-\int _0^{M_k}2f(x)f^\prime (x)dx\\&\qquad =(F_k^+(M_k)-1)f^2(M_k)+\int _0^{M_k}(1-F_k^+(x))2f(x)f^\prime (x)dx+f^2(0)\\&\qquad \le \int _0^{M_k}(1-F_k^+(x))2f(x)f^\prime (x)dx+1\le 2\int _0^{M_k}(1-F_k^+(x))x e^{2\gamma x^2}dx+1. \end{aligned}$$

Applying (19) with $K=3$, we get

$$\begin{aligned}&\int _0^{M_k} (1 - F_k^+(x)) x e^{2\gamma x^2} dx \le 2\int _0^{M_k} x e^{(4\gamma -1+\epsilon )\frac{x^2}{2}} dx\\ {}&\qquad < \frac{2}{4\gamma -1+\epsilon }e^{(4\gamma -1+\epsilon )\frac{(2+\delta )\log \log s_k^2}{2}} \le (\log s_k^2)^{1 - \eta }, \end{aligned}$$

for a suitably small $\eta \in (0,1)$ and large enough *k*, provided $\epsilon ,\delta >0$ are chosen so small that $(4\gamma -1+\epsilon )(2+\delta )<2$. Also, for *k* large enough

$$\begin{aligned}&\int _{|\hat{\xi }_k| \ge M_k} f^2 (\hat{\xi }_k)dP = f^2(M_k)P( |\xi _k| > M_k) \le 2 e^{2\gamma M_k^2} e^{-\frac{M_k^2}{2}(1 - \epsilon ) } < (\log s_k^2)^{1 - \eta } \end{aligned}$$

as before, and thus (23) is proved.

Using Lemma 1, we can complete the proof of Theorem 1 by following the standard path of proving ASCLT’s. Put

27 $$\begin{aligned} \zeta _k= f(\hat{\xi }_k)-Ef(\hat{\xi }_k). \end{aligned}$$

Thus, we have for $k_0\le k\le l$

28 $$\begin{aligned} \Big |E(\zeta _k \zeta _l)\Big | \le 6(\log s_l)^{2 - \eta } (s_k/s_l)^{1/2}, \quad \Big |E(\zeta _k \zeta _l)\Big | \le (\log s_l^2)^{1 - \eta }, \end{aligned}$$

where the second relation follows from (23) and the Cauchy–Schwarz inequality. Let now

29 $$\begin{aligned} d_k = \log (s_{k + 1}^2/s_k^2), \qquad D_n = \sum _{k =1}^n d_k \sim \log s_n^2. \end{aligned}$$

Note that (10) implies that $s_{k+1}^2/s_k^2\rightarrow 1$. Hence, $d_k \sim s_{k+1}^2/s_k^2-1 = \sigma _{k+1}^2/s_k^2$, and thus, in Theorem 1 it suffices to prove relation (11) with the weight $\sigma _{k+1}^2/s_k^2$ replaced by $d_k$ in (29). Clearly

30 $$\begin{aligned} E\biggl (\sum _{k=1}^n d_k \zeta _k\biggr )^2 \le 2 \sum _{1 \le k \le l \le n} d_k d_l \bigl |E(\zeta _k \zeta _l)\bigr |. \end{aligned}$$

Let $0<\delta < \eta /2$. By the first relation of (28), the contribution of those terms on the right-hand side of (30) where for some $\delta >0$

$$\begin{aligned} s_l /s_k \ge \exp (2D_n^\delta ) \end{aligned}$$

is at most

31 $$\begin{aligned}&12 \sum _{1 \le k \le l \le N} d_k d_l (\log s_n^2)^{2 - \eta } \cdot \exp (-D_n^\delta )\le 12 D_n^2 (\log s_n^2)^{2-\eta }\exp (-D_n^\delta ) \nonumber \\&\qquad \sim 12 D_n^2 D_n^{2-\eta }\exp (-D_n^\delta ) =o(D_n^{2-\eta /2}). \end{aligned}$$

On the other hand, since $\frac{s_{k+1}}{s_k}\rightarrow 1$ we have that $L = \sup \limits _{k \ge 1} \frac{s_{k + 1}}{ s_k}<\infty$. Hence, the inequality $s_l / s_k \le \exp (2D_n^\delta )$ implies

$$\begin{aligned} \log s_{l+ 1} - \log s_k \le \log L + 2D_n^\delta , \end{aligned}$$

and thus by the second relation of (28) the contribution of terms on the right-hand side of (30) where $s_l / s_k \le \exp (2D_n^{\delta })$ is at most

32 $$\begin{aligned}&2 \sum _{k = 1}^n d_k \sum _{\{l \ge k :s_l \le s_k \exp (2D_n^\delta )\}} d_l \cdot (\log s_n^2)^{1 - \eta } \nonumber \\&\le 2 (\log s_n^2)^{1 - \eta } \sum _{k = 1}^n d_k \sum _{\{l \ge k:s_l \le s_k \exp (2D_n^\delta )\}} (\log s_{l + 1} - \log s_l) \nonumber \\ {}&\le 2 (\log s_n^2)^{1 - \eta } \sum _{k = 1}^n d_k (\log L + 2D_n^\delta ) \\ {}&\le 5 (\log s_n^2)^{1 - \eta } D_n^\delta \sum _{k = 1}^n d_k \le 6 D_n^{1 - \eta } D_n^{\delta + 1} \nonumber \\ {}&\le 6 D_n^{2 - \eta /2}\nonumber \end{aligned}$$

for $n\ge n_0$ by $\delta <\eta /2$. Thus, using (31) and (32) we get for sufficiently large *n*

$$\begin{aligned} E\biggl (\sum _{k = 1}^n d_k \zeta _k\biggr )^2 \le \text {const} \cdot D_n^{2 - \eta /2}, \end{aligned}$$

and thus, introducing the notation

$$\begin{aligned} T_n = \frac{1}{D_n} \sum _{k=1}^n d_k \zeta _k \end{aligned}$$

we get

$$\begin{aligned} ET_n^2 \le \textrm{const}\cdot D_n^{-\eta /2}. \end{aligned}$$

Since $s_{\ell +1}/s_{\ell }\rightarrow 1$, we can select a sequence $(n_\ell )$ such that $s_{n_\ell }^2 \sim e^{\ell ^{4/\eta }},$ and then the Beppo Levi theorem implies that almost surely $\sum _{\ell =1}^\infty T_{n_\ell }^2 <\infty$ and consequently $T_{n_\ell } \rightarrow 0$ as $\ell \rightarrow \infty$. Hence

33 $$\begin{aligned} \frac{1}{D_n} \sum _{k=1}^n d_k \zeta _k \rightarrow 0 \quad \text {a.s.}\quad (n\rightarrow \infty ) \end{aligned}$$

along the subsequence $(n_\ell )$.

Let $m = \int _\mathbb {R} f(x) d\Phi (x).$ We now show that

34 $$\begin{aligned} {E}f(\hat{\xi }_k) \rightarrow m\quad (k\rightarrow \infty ). \end{aligned}$$

Let $F_k$ denote the distribution function of $\xi _k=S_k/s_k$ and recall that we may fix $f(x)=\exp (\gamma x^2)$. Clearly,

$$\begin{aligned} Ef(\hat{\xi }_k)&=\int _{|x|\le M_k} f(x)dF_k(x)+f(M_k)P(|S_k/s_k|>M_k) \end{aligned}$$

and by (19) and its analogue for the lower tail we obtain that for large enough *k*

$$\begin{aligned} f(M_k)P(|S_k/s_k|>M_k)\le 2\exp (\gamma M_k^2)\exp \left( -\frac{M_k^2}{2}(1-\epsilon )\right) , \end{aligned}$$

which is *o*(1) if $2\gamma <1-\epsilon$. On the other hand, we have that

$$\begin{aligned} m= \int _{|x|\le M_k}f(x) d\Phi (x) +o(1), \end{aligned}$$

and consequently,

35 $$\begin{aligned} Ef(\hat{\xi }_k) -m&= \int _{|x|\le M_k} f(x)d(F_k(x)-\Phi (x)) +o(1) \nonumber \\&=[f(x)(F_k(x)-\Phi (x))]^{M_k}_{-M_k} \\&\quad - 2\gamma \int _{|x|\le M_k}(F_k(x)-\Phi (x)) xe^{\gamma x^2} dx +o(1)\nonumber \end{aligned}$$

using integration by parts. By the CLT (note that the Kolmogorov condition (10) implies the Lindeberg condition), we have

36 $$\begin{aligned} (F_k(x)-\Phi (x)) x e^{\gamma x^2} \rightarrow 0 \end{aligned}$$

for fixed *x* when $k\rightarrow \infty$, and further for $|x|\le M_k$ we have

37 $$\begin{aligned} |F_k(x)-\Phi (x)||x| e^{\gamma x^2}&\le \left( |1-F_k(x)|+|1-\Phi (x)|\right) |x| e^{\gamma x^2} \nonumber \\&\le \left( e^{-\frac{x^2}{2}(1-\epsilon )}+e^{-\frac{x^2}{2}}\right) |x| e^{\gamma x^2} \\&\le 2 |x| e^{-\frac{x^2}{2}(1-\epsilon -2\gamma )}, \nonumber \end{aligned}$$

whose integral in $(-\infty ,\infty )$ is finite when $\epsilon$ is small enough. Thus, the integral in (35) tends to zero by the dominated convergence theorem. The estimates in (37) also show that the first term in (35) tends to 0, and thus, (34) is proved.

We already proved (33) along the sequence $(n_\ell )$; thus, by (34) the relation

$$\begin{aligned} \frac{1}{\log s_n^2} \sum _{k=1}^n d_k f(\hat{\xi }_k) \rightarrow m \quad \text { a.s.} \end{aligned}$$

is also valid along $(n_\ell )$ when $\ell \rightarrow \infty$. By the law of the iterated logarithm for $(X_n)$ (valid by the Kolmogorov condition (10)), we have $P\left( \hat{\xi }_k \ne \xi _k \ \text {i.o.} \right) = 0,$ and thus,

38 $$\begin{aligned} \frac{1}{\log s_n^2} \sum _{k=1}^n d_k f (\xi _k) \rightarrow m \quad \text {a.s.} \end{aligned}$$

along $(n_\ell )$. But since $f > 0$ and by $s_{n_\ell }^2 \sim e^{\ell ^4/\eta }$ we have $\log s_{n_{\ell + 1}}^2 / \log s_{n_\ell }^2 \rightarrow 1$, relation (38) holds along the whole sequence of integers, i.e.,

$$\begin{aligned} \lim _{n\rightarrow \infty } \frac{1}{\log s_n^2} \sum _{k = 1}^n \log \frac{s_{k + 1}^2}{s_k^2} f\left( \frac{S_k}{s_k}\right) = \int _\mathbb {R} f(x) d \Phi (x) \quad \text { a.s.} \end{aligned}$$

Thus, the first half of Theorem 1 is proved.

To prove the second half of Theorem 1, we use an example due to Weiss [20], showing the sharpness of the basic assumption in Kolmogorov’s LIL. Let $X_1,X_2,\ldots$ be independent random variables with

$$\begin{aligned} P( X_{k}=\pm \exp \left( k/\log ^2k\right) )=\frac{\alpha }{2(\log k+\alpha )} \end{aligned}$$

and

$$\begin{aligned} P(X_{k}=0)= 1-\frac{\alpha }{\log k+\alpha }\,. \end{aligned}$$

for a fixed $\alpha >0$. Then simple calculations show (see [20], p. 123) that, using the notations of Theorem 1, we have

39 $$\begin{aligned} \sigma _k^2 \sim \frac{\alpha }{\log k} \exp \left( \frac{2k}{\log ^2 k}\right) , \quad s_k^2 \sim \frac{1}{2}\alpha \exp \left( \frac{2k}{\log ^2 k}\right) \log k \end{aligned}$$

and

40 $$\begin{aligned} \frac{s_k}{(\log \log s_k^2)^{1/2}} \sim \sqrt{\frac{\alpha }{2}} \exp \left( \frac{2k}{\log ^2 k}\right) \end{aligned}$$

as $k\rightarrow \infty$. Thus, (10) holds with *o* replaced by *O*. Also, in [20, Theorem 1] it is shown that if $\alpha$ is sufficiently large, then there is a $\delta >0$ such that almost surely

41 $$\begin{aligned} S_n/s_n >((2+\delta )\log \log s_{n}^{\,2})^{1/2} \end{aligned}$$

for infinitely many *n*. Now let $f(x)=\exp (\gamma x^2)$ where $\frac{1}{2+\delta }<\gamma <1/2$. If we take a fixed *n* which is large enough and satisfies (41), then using (39) and (40) we get that

$$\begin{aligned}&\frac{1}{\log s_{n}^{\,2}}\sum _{k=1}^{n}\frac{\sigma _{k+1}^{\,2}}{s_{k}^{\,2}}\exp \left( \gamma \,\frac{S_{k}^{\,2}}{s_{k}^{\,2}}\right) \ge \frac{1}{\log s_{n}^{\,2}}\frac{\sigma _{n+1}^{\,2}}{s_{n}^{\,2}}\exp \left( \gamma \,\frac{S_{n}^{\,2}}{s_{n}^{\,2}}\right) \\ {}&\ge \text {const}\cdot n^{\gamma (2+\delta )-1} (\log n)^{-2\gamma (2+\delta )}\rightarrow \infty . \end{aligned}$$

This completes the proof of Theorem 1. $\square$

### Proof of Theorem 3

Let, for the purposes of this proof, [*t*] denote the function which equals $s_n^2$ for $s_n^2 \le t < s_{n + 1}^2$ ($n = 1,2,\dots$). Then,

42 $$\begin{aligned} \sum _{k = 1}^n \frac{\sigma _{k+1}^2}{s_k^2} f\left( \frac{S_k}{s_k}\right) = \int \limits _{s_1^2}^{s_{n + 1}^2} \frac{1}{[t]} f \left( \frac{S(t)}{\sqrt{[t]}}\right) dt. \end{aligned}$$

By $s_{n + 1} / s_n \rightarrow 1$ we have $[t] / t \rightarrow 1$ as $t \rightarrow \infty$ and (18) and the LIL for the Wiener process imply $|S(t)| = O((t \log \log t)^{1/2})$ a.s. Thus, by (15) and the mean value theorem we get for $s_n^2 \le t < s_{n + 1}^2$

$$\begin{aligned} \Bigg |\frac{S(t)}{\sqrt{t}} - \frac{S(t)}{\sqrt{[t]}}\Bigg |&\le \text {const} \cdot |S(t)| \frac{t - [t]}{t^{3/2}} \\ {}&\le \text {const} \cdot \frac{(t \log \log t)^{1/2}}{t^{3/2}} \left( s_{n + 1}^2 - s_n^2\right) \\ {}&\le \text { const} \cdot (\log \log s_n^2)^{1/2} \frac{\sigma _n^2}{s_n^2} \rightarrow 0 \ \text { as } \ n \rightarrow \infty . \end{aligned}$$

By our assumption, $f(x)= e^{x^2/2} g(x)$, where $\log g(x)$ is uniformly continuous on $\mathbb R$. Thus, for $n\rightarrow \infty$ we have, uniformly for $s_n^2 \le t < s_{n + 1}^2$,

43 $$\begin{aligned} \frac{f\left( S(t)/\sqrt{t}\right) }{f\left( S(t)/\sqrt{[t]}\right) }= & {} \exp \left( \frac{1}{2} S^2(t) \left( \frac{1}{t} - \frac{1}{[t]}\right) + \log g \left( \frac{S(t)}{\sqrt{t}}\right) - \log g \left( \frac{S(t)}{\sqrt{[t]}}\right) \right) \nonumber \\= & {} \exp \left( O(t \log \log t) \frac{s_{n + 1}^2 - s_n^2}{s_n^4} + o(1)\right) \\= & {} \exp \left( O(\log \log s_n^2) \frac{\sigma _n^2}{s_n^2} + o(1)\right) \nonumber \\= & {} \exp (o(1)) = 1 + o(1).\nonumber \end{aligned}$$

Thus,

44 $$\begin{aligned} \frac{1}{t} f \left( \frac{S(t)}{\sqrt{t}}\right) \sim \frac{1}{[t]} f \left( \frac{S(t)}{\sqrt{[t]}}\right) \ \text { as } \ t \rightarrow \infty \end{aligned}$$,

and consequently, the integral on the right-hand side of (42) is

45 $$\begin{aligned} \sim \int \limits _{s_1^2}^{s_n^2} \frac{1}{t} f\left( \frac{S(t)}{\sqrt{t}}\right) dt. \end{aligned}$$

Now an argument similar to (43) yields, using (18) and the fact that

$$\begin{aligned} |S(t) + W(t)| = O((t \log \log t)^{1/2}) \quad \text {a.s.} \end{aligned}$$

by the LIL,

$$\begin{aligned} \frac{f\left( S(t)/\sqrt{t}\right) }{f\left( W(t)/\sqrt{t}\right) }&= \exp \left( \frac{1}{2} \frac{S^2(t) - W^2(t)}{t} + \log g \left( \frac{S(t)}{\sqrt{t}}\right) - \log g \left( \frac{W(t)}{\sqrt{t}}\right) \right) \\ {}&= \exp \left( \frac{1}{2} \frac{S(t) + W(t)}{\sqrt{t}} \frac{S(t) - W(t)}{\sqrt{t}} + o(1)\right) \\ {}&= \exp \left( O((\log \log t)^{1/2}) o\left( (\log \log t)^{-1/2}\right) + o(1)\right) \\ {}&= \exp (o(1)) = 1 + o(1), \end{aligned}$$

and thus, the integral in (45) is

46 $$\begin{aligned} \sim \int \limits _{s_1^2}^{s_n^2} \frac{1}{t} f \left( \frac{W(t)}{\sqrt{t}}\right) dt. \end{aligned}$$

By the ASCLT for *W*(*t*), we have

47 $$\begin{aligned} \lim _{T\rightarrow \infty } \frac{1}{\log T}\int \limits _A^T \frac{1}{t} f \left( \frac{W(t)}{\sqrt{t}}\right) dt = \int _{\mathbb {R}} f(x) d\Phi (x)\quad \text {a.s.} \end{aligned}$$

for any fixed $A>0$. (This follows from the ergodic theorem by using the substitution $t=e^u$ in the integral on the left-hand side and using the ergodicity of the Ornstein–Uhlenbeck process $e^{-u/2}W(e^u)$.) Thus, the integral in (46), divided by $\log s_n^2$, converges a.s. to $\int _\mathbb {R}f(x) d \Phi (x)$, completing the proof of Theorem 3. $\square$

### Proof of Theorem 2

As in the proof of the first half of Theorem 1, we may assume $f(x)=e^{\gamma x^2/2}$, $0\le \gamma <1/2$. Using (42) and an argument similar to (43) (with $g = 1$ and the constant 1/2 in the second line replaced by $\gamma$), we get

$$\begin{aligned} f \left( \frac{S(t)}{\sqrt{[t]}}\right) \sim f\left( \frac{S(t)}{\sqrt{t}}\right) \ \text { as }\ t \rightarrow \infty . \end{aligned}$$

Thus, for the proof of the first half of Theorem 2 it remains to show

48 $$\begin{aligned} \frac{1}{\log s_n^2} \int \limits _{s_1^2}^{s_n^2} \frac{1}{t} f \left( \frac{S(t)}{\sqrt{t}}\right) dt \rightarrow \int _\mathbb {R} f(x) d\Phi (x). \end{aligned}$$

Fix $0< \varepsilon < 1$. By (16), there exists an a.s. finite random variable $T = T(\varepsilon )$ such that

$$\begin{aligned} \Big | \dfrac{S(t)}{\sqrt{t}} - \dfrac{W(t)}{\sqrt{t}}\Big | \le \varepsilon \quad \text {for} \ t \ge T. \end{aligned}$$

Let

$$\begin{aligned} f_1^{(\varepsilon )}(x)&= {\left\{ \begin{array}{ll} f(|x| - \varepsilon ) &{}\text {if } |x| \ge \varepsilon ,\\ 1 &{}\text { if } |x| < \varepsilon , \end{array}\right. }\\ f_2^{(\varepsilon )}(x)&= f\bigl (|x| + \varepsilon \bigr ). \end{aligned}$$

Clearly, for $t \ge T$ we have

$$\begin{aligned} f \left( \frac{S(t)}{\sqrt{t}}\right) = f\left( \Big |\frac{S(t)}{\sqrt{t}}\Big |\right) \le f \left( \Big |\frac{W(t)}{\sqrt{t}}\Big | + \varepsilon \right) \end{aligned}$$

and for $t \ge T$, $\bigl |W(t) / \sqrt{t}\bigr | \ge \varepsilon$ we have

$$\begin{aligned} f \left( \frac{S(t)}{\sqrt{t}}\right) = f \left( \Big | \frac{S(t)}{\sqrt{t}}\Big |\right) \ge f \left( \Big | \frac{W(t)}{\sqrt{t}}\Big | - \varepsilon \right) . \end{aligned}$$

As $f \ge 1$ everywhere, it follows that for $t \ge T$

49 $$\begin{aligned} f_1^{(\varepsilon )} \left( \frac{W(t)}{\sqrt{t}}\right) \le f \left( \frac{S(t)}{\sqrt{t}}\right) \le f_2^{(\varepsilon )} \left( \frac{W(t)}{\sqrt{t}}\right) . \end{aligned}$$

Now

50 $$\begin{aligned} f_1^{(\varepsilon )}(x) \le f_2^{(\varepsilon )}(x) \le e^{\gamma (|x| + 1)^2} \end{aligned}$$

and by $\gamma < 1/2$ it follows that

51 $$\begin{aligned} \int _\mathbb {R} e^{\gamma (|x| + 1)^2} d \Phi (x) < \infty . \end{aligned}$$

Hence, applying the ASCLT (47) for $f_i^{(\varepsilon )}\bigl (W(t) / \sqrt{t}\bigr )$, $i = 1,2$, it follows from (49) that both the liminf and limsup of

$$\begin{aligned} \frac{1}{\log s_n^2} \int \limits _{T}^{s_n^2} \frac{1}{t} f \left( \frac{S(t)}{\sqrt{t}}\right) dt \end{aligned}$$

as $n \rightarrow \infty$, are between $\int _\mathbb {R} f_1^{(\varepsilon )}(x) d \Phi (x)$ and $\int _\mathbb {R} f_2^{(\varepsilon )}(x) d \Phi (x)$. Obviously, we have $\lim \limits _{\varepsilon \rightarrow 0} f_1^{(\varepsilon )}(x) = \lim \limits _{\varepsilon \rightarrow 0} f_2^{(\varepsilon )}(x) = f(x)$ for every *x*, and thus, (50), (51) and the dominated convergence theorem imply

$$\begin{aligned} \lim \limits _{\varepsilon \rightarrow 0} \int _\mathbb {R} f_i^{(\varepsilon )}(x) d \Phi (x) = \int _\mathbb {R} f(x) d\Phi (x) \quad i = 1,2. \end{aligned}$$

Thus, (48) is proved and the proof of the first half of Theorem 2 is completed.

As we noted after Theorem 2, the first half of the result remains valid for $f(x) = e^{\max \{x^2/2 - cx,0\}}$, $c > 0$. Relation (43) is easily modified in this case, and instead of $0< \varepsilon < 1$, we can use $0< \varepsilon < A$ and then $e^{\gamma (|x| + 1)^2}$ in (50), (51) can be replaced by $f(|x| + A)$. Then, $\int _\mathbb {R} f(|x| + A)d\Phi (x) < \infty$ for sufficiently small *A*.

For the proof of the second half of Theorem 2, we need a lemma.

### Lemma 2

Let $\psi :\ {\mathbb R}^+ \rightarrow {\mathbb R}^+$ be a function with $\lim \limits _{x \rightarrow \infty } \psi (x) = \infty$. Then, there exists a sequence $(X_n)$ of independent r.v.’s with mean zero and finite variances such that setting $S_n = X_1 + \dots + X_n$ we have

52 $$\begin{aligned}{} & {} \qquad \qquad ES_n^2 \sim n\end{aligned}$$

53 $$\begin{aligned}{} & {} \qquad S_n / \sqrt{n} \overset{d}{\rightarrow }\ N(0,1)\end{aligned}$$

54 $$\begin{aligned}{} & {} \bigl |S_n - W(n)\bigr | \le \textrm{const}\,\cdot \, \sqrt{n} \psi (n) \end{aligned}$$

for some Wiener process *W*, but for $f(x) = |x|^p$, $p>2$ and any $\delta > 0$ we have

55 $$\begin{aligned} \int \limits _{|S_n| ~\le ~ \sqrt{(2 + \delta )n \log \log n}} f\left( \frac{S_n}{\sqrt{n}}\right) dP \rightarrow \infty . \end{aligned}$$

### Proof

We use a construction similar to that used in Lifshits [12]. Since there exists a nondecreasing slowly varying function $\psi _1:\ {\mathbb R}^+ \rightarrow {\mathbb R}^+$ with $\psi _1 \le \psi$, $\psi _1(x) = o(\log \log x)^{1/2}$ and $\lim \limits _{x \rightarrow \infty } \psi _1(x) = \infty$, we can assume without loss of generality that $\psi$ is nondecreasing, slowly varying, tends to $+\infty$ and $\psi (x) = o(\log \log x)^{1/2}$. Let $2< \alpha < p$, $n_k = 2^k$ and let $Y_1, Y_2, \dots$ be independent random variables such that

$$\begin{aligned}&P\left( Y_{n_k} = \pm \sqrt{n_k} \psi (n_k)\right) = \psi (n_k)^{-\alpha },\\&P\left( Y_{n_k} = 0\right) = 1 - 2\psi (n_k)^{-\alpha } \end{aligned}$$

and $Y_n \equiv 0$ if $n \ne n_1, n_2, \dots$. Let $S_n^* = Y_1 + \dots + Y_n$. Clearly $EY_{n_k}=0$,

$$\begin{aligned} EY_{n_k}^2 = 2n_k \psi (n_k)^{-(\alpha - 2)} \end{aligned}$$

and thus

$$\begin{aligned} E\bigl (S_{n_k}^*\bigr )^2 = \sum _{j \le k} 2n_j \psi (n_j)^{-(\alpha - 2)} \sim 4n_k \psi (n_k)^{-(\alpha - 2)} \end{aligned}$$

since

$$\begin{aligned} \dfrac{n_{j + 1} \psi (n_{j + 1})^{-(\alpha - 2)}}{n_j \psi (n_j)^{-(\alpha - 2)}} \rightarrow 2 \end{aligned}$$

by the slow variation of $\psi$. Thus,

56 $$\begin{aligned} E\left( \frac{S_{n_k}^*}{\sqrt{n_k}} \right) ^2 \rightarrow 0. \end{aligned}$$

Also, for $k\ge k_0$ we have

57 $$\begin{aligned} \bigl |S_{n_k}^*\bigr | \le \textrm{const}\,\cdot \,\sqrt{n_k} \psi (n_k). \end{aligned}$$

Since $S_n^*$ is a sum of independent, symmetric random variables, we have $P(S_{n_{k-1}}^*\ge 0)\ge 1/2$ and thus

$$\begin{aligned} P(S_{n_k}^* \ge \sqrt{n_k} \psi (n_k))\ge P(S_{n_{k-1}}^*\ge 0)P(Y_{n_k}>0)\ge \frac{1}{2}\psi (n_k)^{-\alpha }. \end{aligned}$$

Moreover, noting that for $n_k \le n < n_{k + 1}$ the partial sum $S_n^*$ does not change, (56) and the latter bound implies that for sufficiently large *n*

58 $$\begin{aligned} E\left( \frac{S_n^*}{\sqrt{n}}\right) ^2 \rightarrow 0,\end{aligned}$$

59 $$\begin{aligned} |S_n^*| \le \textrm{const}\cdot \sqrt{n} \psi (n),\end{aligned}$$

60 $$\begin{aligned} P\left( S_n^* \ge \frac{1}{2} \sqrt{n} \psi (n)\right) \ge \frac{1}{4} \psi (n)^{-\alpha }. \end{aligned}$$

Let now *W* be a Wiener process independent of $\{Y_k, k \ge 1\}$ and set $S_n = W(n) + S_n^*$. Clearly, $\{ S_n, n \ge 1\}$ is the partial sum sequence of a sequence $(X_n)$ of independent random variables with mean zero and finite variances and (58)–(60) show that (52)–(54) hold. To prove (55) with $f(x) = x^p$, let us observe that by (54) and $\psi (x) = o(\log \log x)^{1/2}$, the set of integration in (55) contains for $n \ge n_0$ the set $E_n = \{0 \le W(n) \le \sqrt{n}\}$, whose probability converges to $\Phi (1) - \Phi (0) > 1/4$ as $n \rightarrow \infty$. Thus, by (60) and the independence of *W*(*n*) and $S_n^*$ the probability of the set

$$\begin{aligned} E_n^* = E_n \cap \left\{ S_n^* \ge \frac{1}{2} \sqrt{n} \psi (n)\right\} \end{aligned}$$

exceeds $\frac{1}{16} \psi (n)^{-\alpha }$ for $n \ge n_0$ and on $E_n^*$ we have

$$\begin{aligned} f \left( \frac{S_n}{\sqrt{n}}\right) = \Big | \frac{W(n) + S_n^*}{\sqrt{n}}\Big |^p \ge \Big |\frac{S_n^*}{\sqrt{n}}\Big |^p \ge \left( \frac{1}{2} \psi (n)\right) ^p. \end{aligned}$$

Thus,

$$\begin{aligned} \int \limits _{E_n^*} f \left( \frac{S_n}{\sqrt{n}} \right) dP \ge P(E_n^*) \left( \frac{1}{2} \psi (n)\right) ^p \ge \frac{1}{16\cdot 2^p} (\psi (n))^{p - \alpha } \rightarrow \infty , \end{aligned}$$

proving (55). This completes the proof of Lemma 2.

To prove the second half of Theorem 2, we will follow the argument in the proof of Theorem 1. Let $\delta >0$, $\psi (n)=o(\log \log n)^{1/2}$ and let $(X_n)$ be the sequence provided by Lemma 2. Let $f(x)=e^{\gamma x^2}$ $(0\le \gamma <1/2)$, put $s_k=\sqrt{k}$ and define $M_k$, $\xi _k$, $\xi _{k, l}$, $\hat{\xi }_k$, $\hat{\xi }_{k, l}$ as in the proof of Theorem 1. We claim that Lemma 1 remains valid in the present case. The proof of (22) requires only trivial changes, since the argument there does not use the Kolmogorov condition (10). To prove (23), we set

$$\begin{aligned} B_k = \left\{ |S_k| \le M_k \sqrt{k}\right\} , \quad C_k = \left\{ |S_k| > M_k \sqrt{k} \right\} \end{aligned}$$

and estimate the integral of $f(\hat{\xi }_k)^2$ separately on $B_k$ and $C_k$. We first note that $B_n$ is identical with the set of integration in (55) and by relation (54) on the set $B_k$ we have for sufficiently large *k*

61 $$\begin{aligned} \begin{aligned}&|W(k)|/\sqrt{k} \le |S_k|/\sqrt{k} + \text {const}\,\cdot \, \psi (k) \le |S_k|/\sqrt{k} + \delta (\log \log k)^{1/2} \\&\le M_k + \delta (\log \log k)^{\frac{1}{2}} \le M_k(1 + \delta ), \end{aligned} \end{aligned}$$

and consequently, we have on $B_k$ by (54) and (61),

$$\begin{aligned} S_k^2&\le \left( |W(k)|+\textrm{const}\,\cdot \, \sqrt{k} \psi (k)\right) ^2 \le \left( \Big |W(k)\Big | + \delta (k\log \log k)^{\frac{1}{2}}\right) ^2 \\ {}&\le \left( \Big |W(k)\Big | + \delta \sqrt{k} M_k\right) ^2 \le W(k)^2 + 2 k \delta (1 + \delta ) M_k^2 + \delta ^2 k M_k^2, \end{aligned}$$

i.e.,

$$\begin{aligned} S_k^2/k \le W(k)^2/k + 4\delta M_k^2 + \delta M_k^2 = W(k)^2/k + 5 \delta M_k^2, \end{aligned}$$

whence

$$\begin{aligned} f^2 (S_k/\sqrt{k})&= \exp \left( 2\gamma S_k^2/k\right) \le \exp \left( 2\gamma W(k)^2/k\right) \exp (5\delta (2+\delta )\log \log k) \nonumber \\ {}&\le (\log k)^{15\delta }\exp (2\gamma W(k)^2/k). \end{aligned}$$

Thus, we have to estimate the integral of $\exp (2\gamma W(k)^2/k)$ on the set $B_k$ where, according to the previous relations we have (61), i.e. we get, introducing the standard normal random variable $\zeta = W(k)/\sqrt{k}$,

$$\begin{aligned}&\int \limits _{B_k} e^{2\gamma \frac{W(k)^2}{k}} dP \le \int \limits _{|\zeta |\le M_k(1+\delta )} e^{2\gamma \zeta ^2} dP \\ {}&= \frac{1}{\sqrt{2\pi }} \int \limits _{|x| \le M_k(1 + \delta )} e^{2\gamma x^2} e^{-\frac{x^2}{2}}dx \le \frac{1}{2}\int \limits _{|x| \le M_k (1 + \delta )} e^{\left( 2\gamma - \frac{1}{2}\right) x^2} dx \\&\sim e^{\left( 2\gamma - \frac{1}{2}\right) (1 + \delta )^2 M_k^2} = e^{\left( 2\gamma - \frac{1}{2}\right) (1 + \delta )^2 (2 + \delta ) \log \log k} \\ {}&= (\log k)^{\left( 2\gamma - \frac{1}{2}\right) (1 + \delta )^2 (2 + \delta )}. \end{aligned}$$

Thus, finally we get for sufficiently small $\delta$, using $\gamma <1/2$,

$$\begin{aligned} \int \limits _{B_k} f(\hat{\xi }_k^2) dP \le (\log k)^{\left( 2\gamma - \frac{1}{2}\right) (1 + \delta )^2 (2 + \delta ) + 15\delta } \le (\log k)^{1 - \eta } \end{aligned}$$

for some constant $\eta >0$.

Next we estimate the integral of $f(\hat{\xi }_k^2)$ on the set $C_k$. Let us observe that in view of (54) and $\psi (n)=o(\log \log n)^{1/2}$, on the set $C_k$ we have for sufficiently small $\delta$ and sufficiently large *k*,

$$\begin{aligned}&|W(k)| \ge |S_k| - \frac{\delta }{4} (k \log \log k)^{\frac{1}{2}} \ge \left( \sqrt{2 + \delta } - \frac{\delta }{4}\right) (k \log \log k )^{\frac{1}{2}} \\ {}&\ge \sqrt{2}\left( 1+ \frac{\delta }{20}\right) (k\log \log k)^{\frac{1}{2}} \end{aligned}$$

since

$$\begin{aligned} \sqrt{2 + \delta } - \frac{\delta }{4} \ge \sqrt{2}\left( 1 + \frac{\delta }{20}\right) \end{aligned}$$

for sufficiently small $\delta$ by using the Taylor expansion of $\sqrt{1+\delta /2}$. Thus, using the standard normal variable $\zeta = W(k)/\sqrt{k}$ again, we have

$$\begin{aligned} P(C_k)&\le P \left( \zeta \ge \sqrt{2} \left( 1 + \frac{\delta }{20}\right) (\log \log k)^{\frac{1}{2}}\right) \\ {}&\le \exp \left( -\left( 1 + \frac{\delta }{20}\right) ^2 \log \log k\right) = (\log k)^{-\left( 1 + \frac{\delta }{20}\right) ^2}. \end{aligned}$$

On the other hand, on $C_k$ we have

$$\begin{aligned} f^2 (\hat{\xi }_k)= f^2(M_k) = e^{2\gamma M_k^2} = e^{2\gamma (2 + \delta )\log \log k} = (\log k)^{2\gamma (2 + \delta )}. \end{aligned}$$

Thus, for sufficiently small $\delta$ we get, using $\gamma <1/2$,

$$\begin{aligned} \int \limits _{C_k} f(\hat{\xi }_k)^2 dP&\le (\log k)^{2\gamma (2 + \delta ) - \left( 1 + \frac{\delta }{20}\right) ^2} \le (\log k)^{1 - \eta '} \end{aligned}$$

for some $\eta '>0$. Thus, (23) is proved.

With Lemma 1 established in the present case, we can follow now the proof of Theorem 1 to get, selecting a subsequence $(n_k)$ such that $n_k \sim e^{k^{4/\eta }},$ the relation

62 $$\begin{aligned} \frac{1}{D_n} \sum _{k=1}^n d_k \left( f(\hat{\xi }_k) - E\left( f(\hat{\xi }_k) \right) \right) \rightarrow 0 \quad \text {a.s.} \quad (n\rightarrow \infty ). \end{aligned}$$

along the subsequence $(n_k)$, where $d_k$ and $D_n$ are defined by (29) with $s_k= \sqrt{k}$. However, the argument starting with (29) shows that replacing $d_k$ and $D_n$ in (29) by the analogous quantities $d_k^*\sim d_k$, $D_n^*\sim D_n$, defined with $s_k^*$, relation (33) remains valid and thus (62) holds with the weights based on the true variances of the $X_k$. On the other hand, on the set $B_k=\left\{ |S_k| \le \left( (2+\delta ) k \log \log k\right) ^{1/2}\right\}$ we have $\hat{\xi }_k=\xi _k$, and thus, (55) shows that with $f(x)=|x|^p$, and even more for $f(x)=\exp (\gamma x^2)$, $0<\gamma <1/2$ we have

$$\begin{aligned} \int _{B_k} f(\hat{\xi }_k) dP \rightarrow \infty . \end{aligned}$$

Thus, $E f(\hat{\xi }_k)\rightarrow \infty$ and consequently

$$\begin{aligned} \frac{1}{D_n} \sum _{k=1}^n d_k {E}\left( f(\hat{\xi }_k) \right) \rightarrow 0 \quad \text {a.s.}\quad (n\rightarrow \infty ). \end{aligned}$$

Thus, relation (62) yields

63 $$\begin{aligned} \frac{1}{D_n} \sum _{k=1}^n d_k f(\hat{\xi }_k) \rightarrow \infty \quad \text {a.s.} \end{aligned}$$

along the subsequence $(n_k)$. But

$$\begin{aligned} P\left\{ \xi _k \ne \hat{\xi }_k \ \text {i.o.}\right\} = 0, \end{aligned}$$

by the law of the iterated logarithm (implied by (54)), and thus, (63) yields

$$\begin{aligned} \limsup _{N\rightarrow \infty } \frac{1}{D_n} \sum _{k = 1}^n d_k f\left( \xi _k \right) =+\infty \quad \text { a.s.}, \end{aligned}$$

completing the proof of the second half of Theorem 2. $\square$

## Data Availability

The data that support the findings of this study are available from the corresponding author upon reasonable request.
